# PON1 haplotypes show genotype-dependent associations with dysglycemia and metabolic liver risk beyond paraoxonase activity

**DOI:** 10.3389/fendo.2026.1870186

**Published:** 2026-07-07

**Authors:** Laura Batista-Herrera, Maria João Meneses, Rogério T. Ribeiro, Luís Gardete-Correia, João F. Raposo, José Manuel Boavida, Carlos Penha-Gonçalves, Maria Paula Macedo

**Affiliations:** 1 iNOVA4Health, NOVA Medical School, Faculdade de Ciências Médicas (NMS, FCM), Universidade NOVA de Lisboa, Lisboa, Portugal; 2 APDP – Diabetes Portugal, Education and Research Center, Lisbon, Portugal; 3 ProRegeM PhD Program, NOVA Medical School (NMS), Universidade Nova de Lisboa, Lisbon, Portugal; 4 Portuguese Society of Diabetology, Lisbon, Portugal; 5 Global Health and Tropical Medicine (GHTM), Associated Laboratory in Translation and Innovation Towards Global Health (LA-REAL), Instituto de Higiene e Medicina Tropical (IHMT), Universidade NOVA de Lisboa, Lisbon, Portugal

**Keywords:** dysglycemia, genetic epidemiology, metabolic liver disease, oxidative stress, paraoxonase, PON1

## Abstract

**Introduction:**

Paraoxonase 1 (PON1) is a liver-derived HDL-associated enzyme with key antioxidant functions implicated in cardiometabolic disease. Genetic variation in PON1 strongly influences enzyme activity; however, whether specific genetic configurations contribute to dysglycemia and metabolic liver risk beyond enzymatic activity remains unclear.

**Methods:**

We analyzed 922 individuals from the PREVADIAB2 cohort to investigate the relationship between PON1 genetic variation, serum paraoxonase (PONase) activity, and dysmetabolic phenotypes. Genetic determinants of PONase activity were identified using genome-wide analysis. Independent variants were combined into haplotypes, and their associations with dysglycemia and metabolic liver risk (Fibrotic NASH Index, FNI) were assessed in individuals aged >55 years.

**Results:**

Two independent PON1 variants—rs2057681 (in strong linkage disequilibrium with Q192R) and the promoter variant rs854572—were identified as major determinants of PONase activity. Haplotype analysis revealed that promoter–transcribed region combinations exert graded effects on enzyme activity. Importantly, these genetic configurations were differentially associated with dysglycemia and metabolic liver risk in a genotype-dependent manner. In carriers of the rs2057681 G allele, the C–A haplotype was associated with lower risk of dysglycemia and elevated FNI, whereas in rs2057681 AA homozygotes the same haplotype showed an opposite association with metabolic liver risk. Notably, despite strong genetic effects on PONase activity, enzyme activity itself was not directly associated with dysmetabolic phenotypes.

**Discussion:**

PON1 genetic architecture, defined by promoter– transcribed region interactions, is associated with dysglycemia and metabolic liver risk in a genotype-dependent manner beyond steady-state enzyme activity. These findings provide insight into the genetic regulation of metabolic risk and may help explain inconsistent associations of PON1 variants in cardiometabolic disease.

## Graphical Abstract

**Figure:**
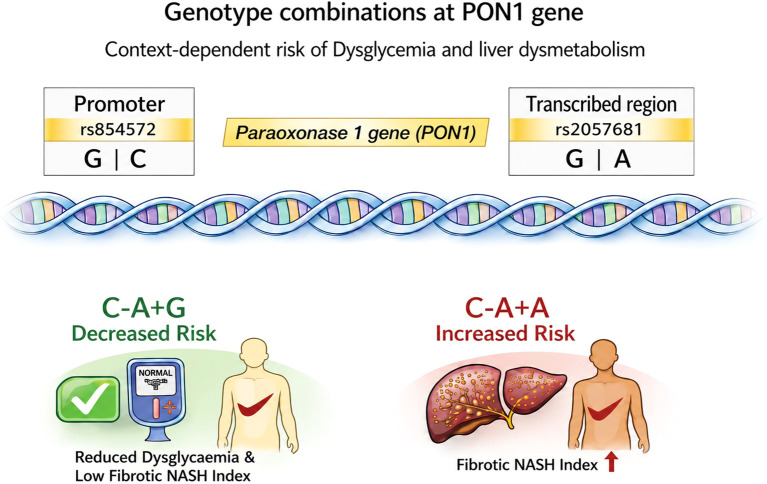

## Introduction

Cardiometabolic disorders, including type 2 diabetes and metabolic dysfunction–associated steatotic liver disease (MASLD), arise from complex interactions between metabolic imbalance, chronic inflammation, and oxidative stress (1, 2). These conditions frequently coexist and share common pathophysiological mechanisms, in which lipid dysregulation and impaired glucose homeostasis reinforce each other and contribute to progressive organ damage (3–5). In particular, oxidative stress plays a central role by promoting lipid peroxidation, mitochondrial dysfunction, and inflammatory signaling, ultimately impairing insulin sensitivity and metabolic control (2, 6, 7).

Paraoxonase 1 (PON1) is a liver-derived enzyme associated with high-density lipoprotein (HDL) particles that contributes to antioxidant defense through the hydrolysis of oxidized lipids and lipid peroxides (8, 9). Through this activity, PON1 is implicated in the regulation of lipid oxidation, endothelial function, and inflammatory responses (10, 11). Reduced PON1 activity has been associated with increased cardiometabolic risk, including atherosclerosis, type 2 diabetes, and MASLD, supporting its role as a protective factor in oxidative stress–related conditions (12).

Interindividual variability in PON1 activity is largely determined by genetic variation within the PON1 gene. Functional polymorphisms in both transcribed and promoter regions influence enzyme expression, stability, and substrate specificity. Among the most extensively studied are the promoter region variant −108C>T (rs705379) and the coding region variants Q192R (rs662) and L55M (rs854560) (13–15). The −108C allele has been associated with higher transcriptional activity and increased serum PON1 levels, presumably via enhanced affinity for transcription factor binding sites. Similarly, the Q192R and L55M variants modulate enzymatic activity and stability, with the 192Q isoform displaying greater degradation efficacy of lipid peroxides and the 55L variant showing higher plasma protein concentrations (13–18). However, cumulative evidence suggests that the biological and clinical relevance of PON1 require investigating the relationship between individual PON1 variants and cardiometabolic diseases as single-variant approaches do not fully capture the functional complexity of the PON1 system (19–21).

Emerging evidence indicates that the combined effects of multiple genetic variants, including interactions between regulatory and coding regions, may better reflect the biological regulation of PON1 (22, 23). Such combinatorial genetic configurations may influence enzyme function and disease susceptibility in a context-dependent manner that is not adequately represented by enzyme activity measurements alone. Understanding how genetic variation at the PON1 locus contributes to dysmetabolic phenotypes may provide insight into the interplay between oxidative stress and metabolic disease.

In this study, we investigated the genetic determinants of serum PON1 paraoxonase activity and examined how specific promoter–transcribed region genetic configurations associate with dysglycemia and also metabolic liver risk in a population-based cohort. We further assessed whether these associations are explained by enzyme activity or reflect broader genetic influences on cardiometabolic susceptibility.

## Materials and methods

### Ethical permits and study cohort

All participants were volunteers and provided written informed consent for inclusion in the PREVADIAB2 study (24). The protocol followed the principles of the Declaration of Helsinki and received approval from the Ethics Committees of Associação Protectora dos Diabéticos de Portugal (APDP Diabetes Portugal (900/2013)) and NOVA Medical School (CEFCM/02/2020), as well as from the Portuguese Data Protection Authority (permit no. 3228/2013). The study population was drawn from the PREVADIAB2 cohort and initially comprised 924 genotyped subjects.

### Biochemical assessments

Determination of glycemic status was based on glucose measurements in blood samples collected after a 12-hour overnight fast and during a standard 75 g oral glucose tolerance test (OGTT) at 0, 30, and 120 minutes. Plasma glucose was quantified by the glucose oxidase method (Olympus AU640, Beckman Coulter). Glycemic status was defined according to WHO/International Diabetes Federation (IDF) criteria (25). The Fibrotic NASH Index (FNI) scoring tool (26, 27) was applied to estimate metabolic liver risk, based on serum measurements of aspartate aminotransferase (AST), high-density lipoprotein (HDL) cholesterol using enzymatic methods in an automated analyzer (Olympus AU640, Beckman Coulter) and glycated hemoglobin (HbA1c) measured by high-performance liquid chromatography with boronate affinity (Menarini Premier Hb 9210).

### PON1 activity analysis

Following Batuca et al. (28), serum paraoxonase (PONase) activity was assessed using a substrate mixture containing paraoxon (1.0 mM; Sigma-Aldrich) freshly prepared in 50 mM glycine buffer containing 1 mM calcium chloride (pH 10.5). For each sample, 10 μL of serum were added to 290 μL of substrate mixture and incubated at 37 °C in 96-well plates. The release of *p*-nitrophenol was measured at 412 nm between 10 and 50 minutes of incubation and activity was expressed as units per mL serum.

### Genetic data and analysis

Genomic DNA was genotyped using the Axiom™ HGCoV2_1 array (Thermo Fisher Scientific). Variant positions were aligned to the GRCh38/hg38 reference genome and annotated using dbSNP v151 and the 1000 Genomes Project Phase 3 reference panel.

Standard quality control procedures were applied using PLINK v1.9, including exclusion of individuals and variants with >5% missingness and variants deviating from Hardy–Weinberg equilibrium (P < 1×10⁻^7^). Variants with minor allele frequency <10% were excluded to focus on common variants. Population structure was assessed by principal component analysis using LD-pruned autosomal variants. Individuals deviating by more than 3 standard deviations from the cohort mean on PC1 or PC2 were considered ancestry outliers and excluded. Genetic relatedness was assessed using identity-by-descent estimates (PLINK v1.9) and removal of ancestry outliers used a $\hat{\text{π}}$>0.175 threshold, corresponding to approximately second-degree relationships. After initial genotype QC, 922 individuals remained. PCA-based ancestry filtering further excluded 15 individuals (**Supplementary Figure 3**) while relatedness filtering excluded 28 additional individuals. After ancestry and relatedness filtering, 879 individuals and approximately 240,000 variants were retained for analysis.

To identify genetic determinants of serum PONase activity, we performed genome-wide association analyses (PLINK v1.9) using linear regression models adjusted for age, sex, and body mass index. Genomic inflation was assessed using the lambda statistic. Genome-wide significance was assessed using both Bonferroni correction and false discovery rate (FDR) adjustment (Benjamini–Hochberg method). Bonferroni-adjusted P values and FDR-adjusted q values are reported for genome-wide analyses.

Given the strong signal observed at the PON1 locus, subsequent analyses focused on this region to characterize independent genetic effects. Conditional association analysis was performed to identify variants independently associated with PONase activity. Conditional and joint multiple-SNP analysis was performed using GCTA-COJO with the PREVADIAB2 genotype data as the LD reference panel to evaluate whether different association signals at the PON1 locus rs2057681 and rs854572 represented independent effects. Based on these results, two variants representing coding-region and promoter-region effects (rs2057681 tagging the Q192R-linked coding signal and rs854572, respectively) were selected for downstream analyses.

### Haplotype construction and genetic modeling

Haplotypes were constructed from rs2057681 and rs854572 using phased genotype data generated with Beagle v5.5. Four haplotypes were observed and analyzed (G–A, C–A, G–G, and C–G; first allele rs854572, second allele rs2057681). Given the dominant effect of rs2057681 on PONase activity, analyses of clinical outcomes were stratified by rs2057681 genotype to evaluate whether the effect of the promoter variant rs854572 differed according to the genetic background linked to the coding region. This approach was used to investigate genotype-context interactions between regulatory and transcribed region variants.

### Clinical outcomes

Glycemic status and metabolic liver risk were defined as described above. The study population was grouped into normoglycemic (n = 638) or dysglycemic subjects (n = 261, including prediabetes and diabetes), and into low (FNI ≤0.1, n = 525) or higher (FNI >0.1, n = 394) metabolic liver risk. Analyses of dysglycemia and metabolic liver risk were restricted to individuals aged over 55 years to focus on clinically established metabolic phenotypes and reduce heterogeneity related to early or transient metabolic alterations.

### Statistical analysis

Associations with continuous variables (serum PONase activity) were assessed using non-parametric tests, including the Kruskal–Wallis test with Dunn’s correction for multiple comparisons, performed in GraphPad Prism (v10.2).

Associations with dysmetabolic outcomes (dysglycemia and elevated FNI) were evaluated using Poisson regression models with log link and robust variance estimation as previously described by Zou (29) to obtain adjusted relative risks (RR) and 95% confidence intervals. Models were adjusted for age, sex, and body mass index. Analyses were stratified by rs2057681 genotype to assess genotype-context–dependent associations. A two-sided P value <0.05 was considered statistically significant. Poisson regression analyses were performed using the statsmodels library (v0.14.4) in Python (v3.12.3) adjusting for age, sex and BMI. Sensitivity analyses additionally considered available clinical covariates, including smoking, HDL cholesterol, AST, hypertension, and triglycerides.

## Results

### Genetic determinants of serum PON1 activity

Genome-wide analysis was performed to identify genetic determinants of serum paraoxonase (PONase) activity in the PREVADIAB2 cohort. After quality control, approximately 240,000 single nucleotide polymorphisms (SNPs) were analyzed in 922 individuals with available PONase activity measurements.

**Figure 1:**
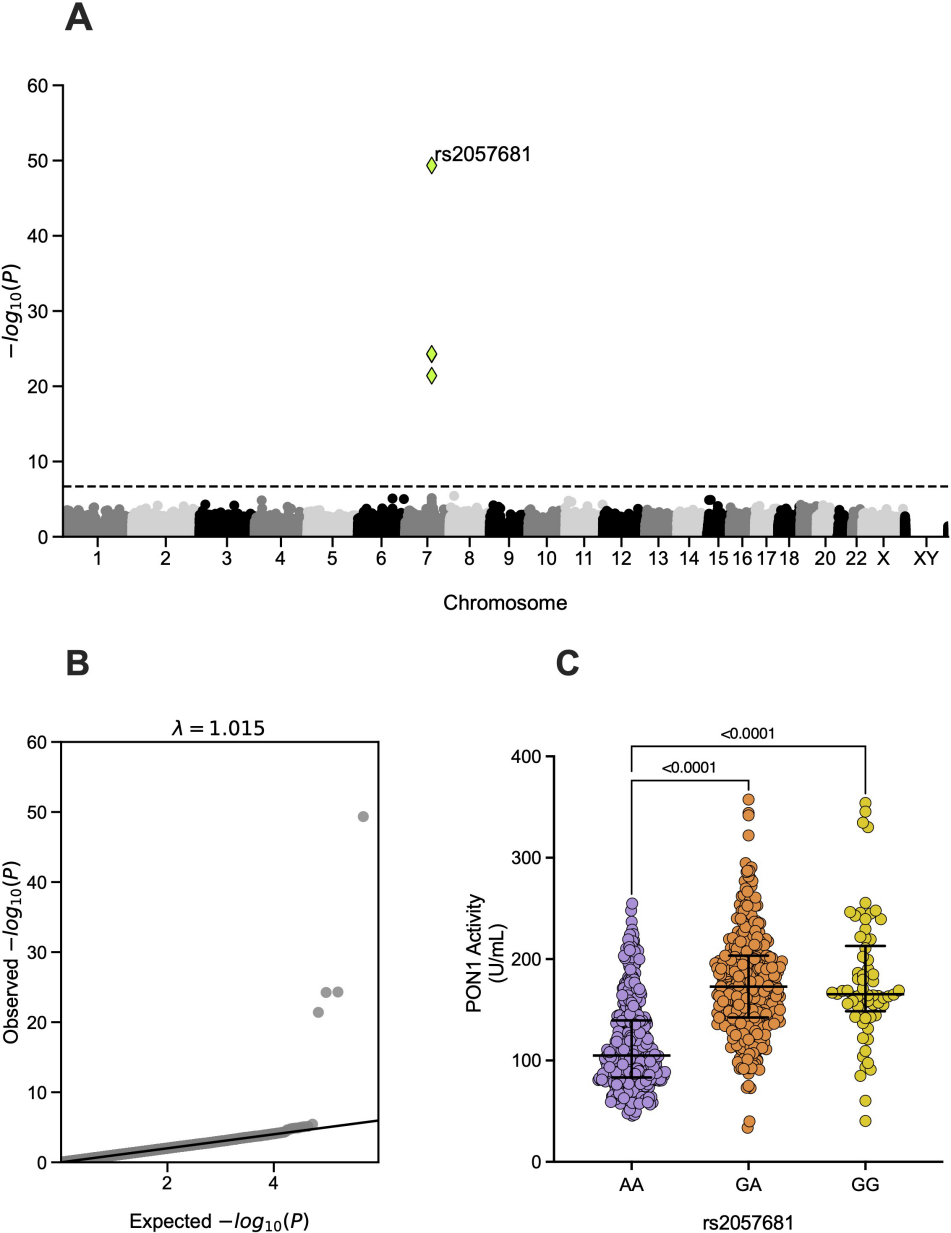
Genome-wide analysis of serum PONase activity in the PREVADIAB2 cohort. **(A)** Manhattan plot of genome-wide analysis for serum PONase activity in the PREVADIAB2 cohort (N = 879). A total of 240,830 SNPs were tested using a linear additive model adjusted for age, sex, and BMI. The x-axis represents genomic position by chromosome, and the y-axis shows –log_10_(P values). The horizontal dashed line indicates the genome-wide significance threshold (P = 2.07 × 10⁻^7^). Seven significant SNPs are highlighted as green diamonds. The strongest association was observed at rs2057681 (Padj. Bonf. = 3.20 × 10^-54^), located in the PON1 region on chromosome 7. **(B)** Quantile–quantile (QQ) plot showing observed versus expected –log_10_(P values) under the null hypothesis. The genomic inflation factor (λ = 1.015) indicates minimal inflation. **(C)** Genetic effect of the lead SNP rs2057681 on serum PONase activity. Genotypes (AA, GA, GG) are shown on the x-axis and enzyme activity on the y-axis. Data points represent individual values; horizontal lines and error bars indicate median and interquartile range. Genotype group sizes were AA = 390, AG = 367, and GG = 66. The G allele was associated with increased enzyme activity in a dominant manner.

Following adjustment for age, sex, and body mass index, a major locus on chromosome 7 emerged as the principal determinant of serum PONase activity (**Figures 1A, B**). Seven SNPs spanning a 22.2 kb region within the PON1 gene achieved genome-wide significance under a dominant genetic model after correction for multiple testing (**Table 1**). These variants are in strong linkage disequilibrium (LD) and define a distinct LD block within the PON1 locus (**Supplementary Figure 1**). This region lies within a cluster of 27 genotyped SNPs spanning the PON1, PON2, and PON3 genes on chromosome 7. These findings are consistent with previous reports identifying PON1 genetic variation as a major determinant of serum PON1 enzymatic activity (16).

**Table 1:** Genome-wide significant variants associated with PONase serum activity: Seven SNPs map within the PON1 gene region.

CHR	BP	Ref. SNP	Ref. All.	Gene context	n	HWE	MAF	BETA	STAT	*P*	*P* Adj. Bonf.	P Adj. FDR
7	95294544	rs854547	G	Downstream	784	0.77	0.38	45.56	11.78	1.36E-29	3.84E-24	7.68E-25
7	95301079	rs854555	A	Intron	785	0.72	0.37	46.83	12.23	1.34E-31	3.77E-26	9.43E-27
7	95305887	rs3917549	T	Intron	785	0.43	0.18	45.84	11.39	6.29E-28	1.78E-22	2.96E-23
7	95308134	rs662	C	Exon 6 (Q192R)	784	0.38	0.31	61.41	17.69	4.10E-59	1.16E-53	3.86E-54
7	95308945	rs2057681	G	Intron	785	0.43	0.31	61.66	17.79	1.13E-59	3.20E-54	2.26E-54
7	95311726	rs1157745	T	Intron	783	0.39	0.31	61.73	17.77	1.60E-59	4.52E-54	2.26E-54
7	95316772	rs854560	T	Exon 3 (L55M)	784	0.94	0.39	-29.04	-7.06	3.61E-12	1.02E-06	1.46E-07

Results of linear regression analysis using a dominant genetic model, adjusted for age, sex, and BMI. CHR: chromosome; BP: base pair position (GRCh38.p14); Ref. SNP: reference SNP ID; Ref. All.: reference allele; n: number of individuals; HWE: Hardy–Weinberg equilibrium p-value; MAF: minor allele frequency; BETA: regression coefficient; STAT: t-statistic; P: nominal p-value; P Adj. Bonf.: Bonferroni-corrected p-value; P Adj. FDR: False Discovery Rate adjusted p-value (Benjamini–Hochberg method).

To evaluate whether metabolic status influenced these associations, analyses were stratified by glycemic status (normoglycemia vs dysglycemia) and metabolic liver status (FNI ≤0.1 vs FNI >0.1). The association between PON1 locus variants and PONase activity remained consistent across strata (**Supplementary Figure 2**; **Supplementary Table 1**), indicating that enzyme activity is primarily genetically determined and not substantially modified by dysglycemia or metabolic liver risk.

The strongest association with PONase activity was observed at rs2057681, which reached genome-wide significance under both additive (Padj = 3.99 × 10⁻^48^) and dominant (Padj = 2.04 × 10⁻^57^) models (**Supplementary Table 2**). The G allele was associated with increased PONase activity in a dominant manner (**Figure 1C**). This intronic variant maps within the transcribed PON1 gene body region and is in near-complete linkage disequilibrium (r² = 0.995) with rs662 (Q192R), a well-established functional coding variant that strongly influences PON1 enzymatic activity (**Supplementary Table 3**).

**Figure 2:**
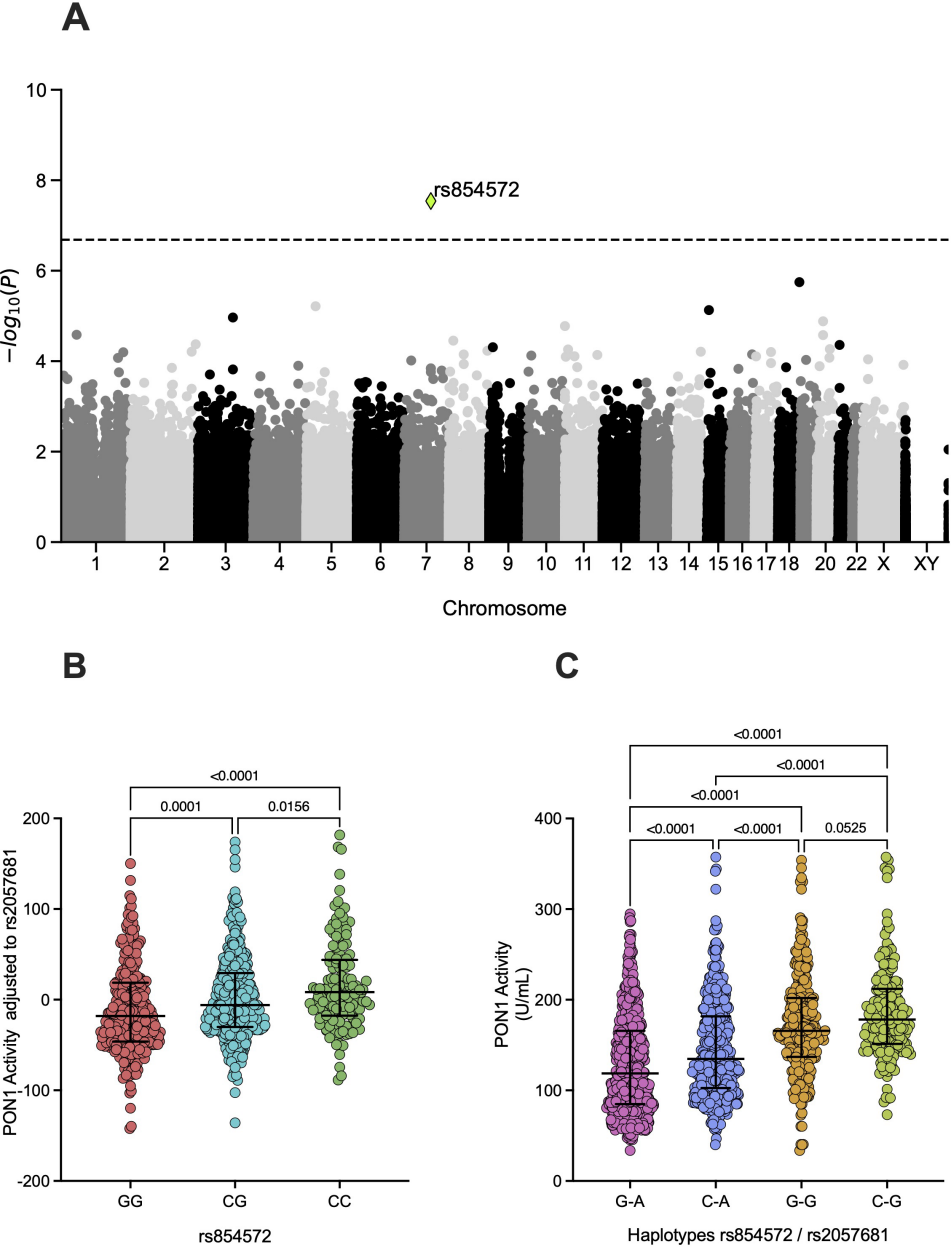
Conditional analysis and combinatorial genetic effects on serum PONase activity. **(A)** Manhattan plot of conditional genome-wide analysis for serum PONase activity in the PREVADIAB2 cohort after adjustment for rs2057681. The analysis included 879 individuals and 240,830 SNPs tested using a linear additive model. The x-axis shows chromosomal position and the y-axis shows –log_10_(P value). The horizontal line marks the genome-wide significance threshold (P = 2.07 × 10⁻^7^). After conditioning, rs854572 was the only SNP exceeding this threshold. **(B)** Residual serum PONase activity according to rs854572 genotype (GG, CG, CC) after adjustment for rs2057681 genotype. Violin plots with overlaid box plots show the distribution, median, and interquartile range of residual PONase activity. Genotype group sizes were GG = 302, CG = 377, and CC = 143. **(C)** Association of PON1 haplotypes with serum PONase activity. Haplotypes were constructed from rs854572 and rs2057681, yielding four allelic combinations: G–A, C–A, G–G, and C–G (first allele = rs854572, second allele = rs2057681). Violin plots with overlaid box plots show the distribution, median, and interquartile range of PONase activity in each haplotype group. Haplotype group sizes were G–A = 673, C–A = 474, G–G = 310, and C–G = 189. Statistical comparisons in panels B and C were performed using the Kruskal–Wallis test with Dunn’s correction.

Conditional analysis identified rs854572, located in the PON1 promoter region, as an independent signal associated with PONase activity (P = 2.89 × 10^–8^; Padj. Bonf = 6.93 × 10^–7^). The C allele was associated with increased enzyme activity (**Figure 2**; **Supplementary Table 2**). This variant is not in linkage disequilibrium with rs2057681 and lies in a neighboring LD block (**Supplementary Figure 1**; **Supplementary Table 3**). GCTA-COJO analysis confirmed two independent association signals at the PON1 locus: rs2057681 remained strongly associated with PONase activity after conditioning on rs854572 (joint P = 2.27 × 10^–43^), and rs854572 remained associated after conditioning on rs2057681 (joint P = 1.24 × 10^–5^) (**Supplementary Table 4**).

Its association with PONase activity is consistent with previously reported regulatory effects on PON1 expression (17).

### PON1 genetic configurations and dysmetabolic phenotypes

To investigate how independent PON1 genetic signals jointly influence enzyme activity and dysmetabolic outcomes, we focused on rs854572 and rs2057681, representing promoter-region and coding-region effects, respectively.

Haplotype analysis identified four rs854572–rs2057681 combinations [G–A, C–A, G–G, and C–G (**Supplementary Table 5**)]. These haplotypes showed a graded association with serum PONase activity. The C–G haplotype was associated with the highest activity, whereas the G–A haplotype showed the lowest. The G–G haplotype conferred higher activity than the C–A haplotype, indicating a stronger contribution of rs2057681 genotype to enzyme activity (**Figure 2C**).

Associations with dysmetabolic outcomes were evaluated in individuals aged over 55 years. Given the dominant effect of rs2057681 on enzyme activity, analyses were stratified by rs2057681 genotype to assess genotype-context–dependent associations. Adjusted relative risks (RR) were estimated using Poisson regression models controlling for age, sex, and body mass index.

**Figure 3:**
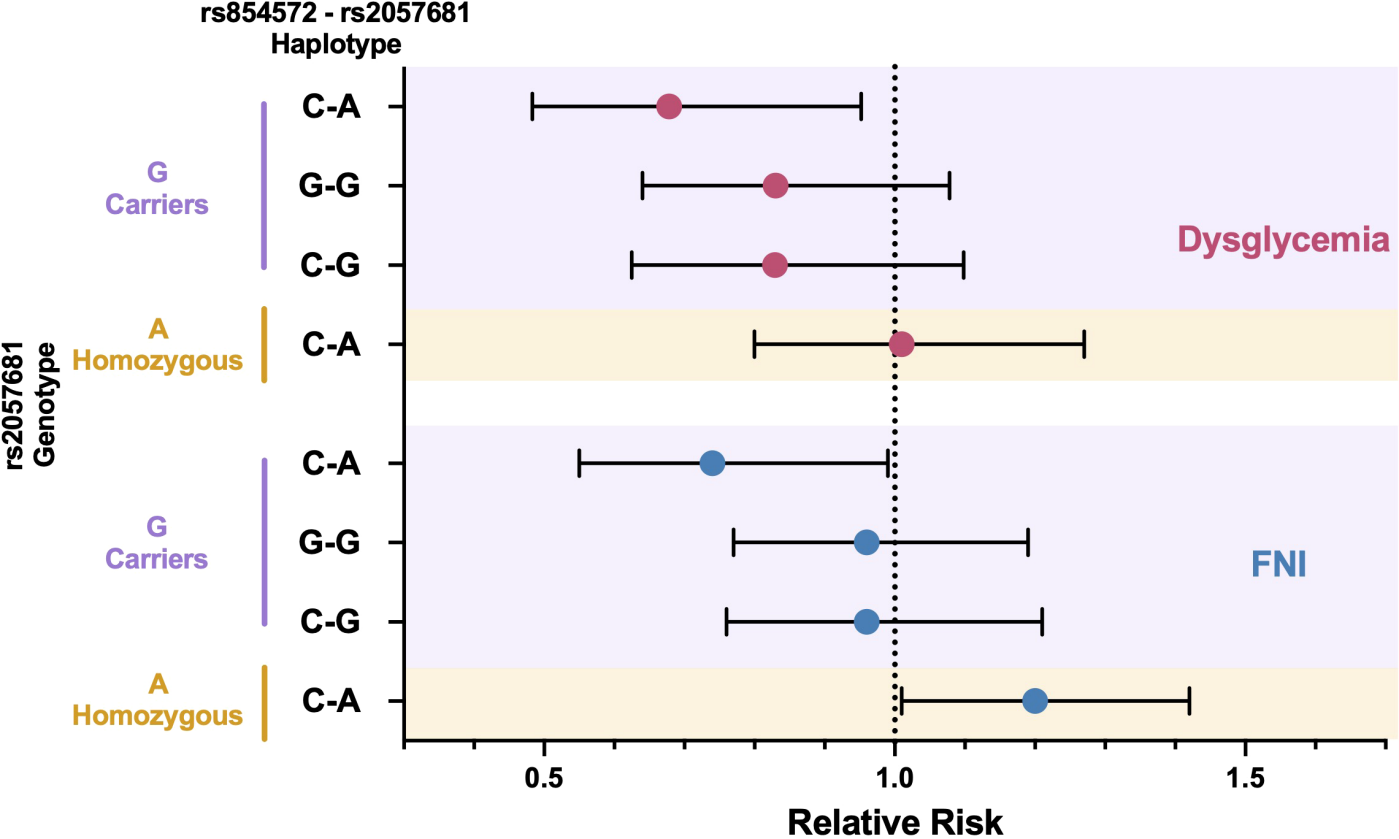
PON1 promoter– transcribed region haplotypic combinations associated with dysglycemia and metabolic liver risk. Relative risk estimates for dysglycemia (red dots) and elevated FNI (blue dots) associated with rs854572 (promoter)–rs2057681 (transcribed region) haplotypic combinations (C–A, G–G, and C–G), using the G–A haplotype as reference. Estimates were obtained in individuals aged over 55 years and stratified by rs2057681 genotype background (GA/GG or AA), adjusted for age, sex, and BMI as described in **Tables 2** and **3**. Error bars represent 95% confidence intervals.

Among carriers of at least one G allele (GA or GG genotypes), the G–A haplotype was associated with the highest prevalence of both dysglycemia and elevated FNI and was therefore used as the reference group. Within this genetic background, carriers of the C–A haplotype showed lower prevalence of dysglycemia (adjusted RR 0.68, 95% CI 0.48–0.95; P = 0.025) and metabolic liver risk (adjusted RR 0.74, 95% CI 0.55–0.99; P = 0.046) compared with G–A carriers (**Tables 2**, **3**; **Figure 3**). In contrast, the G–G and C–G haplotypes were not significantly associated with dysglycemia or metabolic liver risk despite being linked to higher PONase activity.

**Table 2:** PON1 haplotypes association with dysglycemia, stratified by rs2057681 genotype.

Haplotype	Alelle rs854572	Alelle rs2057681	n	Dysglycemia (%)	RR (adj.)	95 % CI	P value
rs2057681 GA/GG carriers							
G–A (ref)	G	A	147	42.2%	1.00 (ref)	—	—
C–A	C	A	114	27.2%	**0.68**	0.48 – 0.95	**0.025**
G–G	G	G	222	32.9%	0.83	0.64 – 1.08	0.162
C–G	C	G	135	35.6%	0.83	0.63 – 1.10	0.190
rs2057681 AA homozygotes							
G–A (ref)	G	A	345	32.6%	1.00 (ref)	—	—
C–A	C	A	241	32.6%	1.01	0.80 – 1.27	0.953

Haplotype: rs854572-rs2057681 haplotypes; n: haplotype count; Adjusted relative risks (RR) were estimated using Poisson regression models with log link, adjusting for age, sex, and body mass index. Statistically significant results (P < 0.05) are shown in bold.

**Table 3:** PON1 haplotypes association with metabolic liver risk (FNI >0.1), stratified by rs2057681 genotype.

Haplotype	Alelle rs854572	Alelle rs2057681	n	FNI (>0.1) (%)	RR (adj.)	95 % CI	P value
rs2057681 GA/GG carriers							
G–A (ref)	G	A	149	47.0%	1.00 (ref)	—	—
C–A	C	A	115	33.0%	**0.74**	0.55 – 0.99	**0.046**
G–G	G	G	224	43.3%	0.96	0.77 – 1.19	0.683
C–G	C	G	138	45.7%	0.96	0.76 – 1.21	0.720
rs2057681 AA homozygotes							
G–A (ref)	G	A	344	42.4%	1.00 (ref)	—	—
C–A	C	A	240	51.7%	**1.2**	1.01 – 1.42	**0.035**

Haplotype: rs854572-rs2057681 haplotypes; n: haplotype count; Adjusted relative risks (RR) were estimated using Poisson regression models with log link, adjusting for age, sex, and body mass index. Statistically significant results (P < 0.05) are shown in bold.

In individuals homozygous for the A allele (AA genotype), a distinct pattern was observed. The C–A haplotype was not associated with dysglycemia as compared with the G–A haplotype (adjusted RR 1.01, 95% CI 0.80–1.27; P = 0.953), whereas a modest association with metabolic liver risk was observed (adjusted RR 1.20, 95% CI 1.01–1.42; P = 0.035) (**Tables 2**, **3**). Thus, the same promoter– transcribed region combination (C–A) showed divergent associations across dysmetabolic outcomes depending on rs2057681 genotype background.

Sensitivity analyses including additional available clinical covariates, showed that the direction of the main C–A haplotype associations was generally preserved, although some estimates were attenuated after adjustment for metabolic variables closely related to the outcomes (**Supplementary Table 6**). In particular, adjustment for AST attenuated the association with elevated FNI, consistent with AST being a component of the FNI score.

Overall, these findings indicate that PON1 promoter– transcribed region genotype combinations are associated with dysglycemia and metabolic liver risk in a genotype-dependent manner. Although these genetic configurations strongly influence serum PONase activity, their associations with dysmetabolic phenotypes are not directly explained by steady-state serum PONase activity.

## Discussion

In this study, we investigated the contribution of genetic variation at the PON1 locus to enzyme activity and dysmetabolic phenotypes. We identified two independent signals, represented by rs2057681 and rs854572, that jointly influence serum PONase activity. Importantly, we observed that specific combinations of promoter– transcribed region variants were differentially associated with dysglycemia and metabolic liver risk, depending on rs2057681 genotype background.

Consistent with previous reports (30, 31), rs2057681 showed the strongest association with serum PONase activity, likely reflecting its linkage with the functional Q192R polymorphism. The 192R isoform has been associated with increased hydrolysis of paraoxon but altered activity toward physiological substrates, including lipid peroxides, which may influence oxidative stress responses in a context-dependent manner (31, 32). Notably, the frequency and disease associations of Q192R alleles vary across populations, with divergent effects reported in different ethnic groups (19, 33). These observations suggest that the relationship between PON1 genetic variation, enzyme function, and disease risk is complex and may depend on additional genetic and environmental modifiers.

Our findings extend this concept by showing that promoter and coding-region variants act in combination rather than in isolation. Conditional analyses identified rs854572 as an independent contributor to PONase activity, supporting a regulatory role for promoter-region variation (34). Although rs854572 has limited direct functional annotation, its linkage with known promoter variants and prior associations with lipid traits and vascular outcomes are consistent with genetically controlled regulatory effects (35).

We also showed that rs854572–rs2057681 haplotypes showed a graded relationship with enzyme activity, but their associations with dysglycemia and metabolic liver risk followed a distinct pattern that depended on the rs2057681 genotype context (**Figures 2C**, **3**). In individuals carrying at least one rs2057681 G allele, the C–A haplotype was associated with lower prevalence of dysglycemia and metabolic liver risk. In contrast, among rs2057681 AA homozygotes, this pattern was not observed, and the same haplotype showed no protective association with dysglycemia and a modest association with metabolic liver risk. These findings highlight the importance of PON1 genetic context in shaping disease associations.

The genotype-dependent pattern observed for the C–A haplotype may reflect the distinct effects of the two PON1 signals. rs2057681 tags the Q192R-linked signal and was the strongest determinant of measured paraoxonase activity, whereas rs854572 represents an independent promoter-region signal. However, paraoxonase activity measured with paraoxon may not fully capture PON1 functions most relevant to metabolic disease, including HDL-associated antioxidant activity and lipid-peroxide metabolism. Thus, the C–A haplotype may have different biological consequences depending on the rs2057681/Q192R-linked background in which it occurs. These observations suggest that promoter variation may not act uniformly across all PON1 genetic backgrounds but instead modifies dysmetabolic associations in a genotype-dependent manner.

This genotype-dependent pattern may help explain inconsistencies in the literature regarding associations between PON1 variants and cardiometabolic outcomes. Previous studies have reported heterogeneous and sometimes contradictory findings, which may reflect differences in underlying PON1 genetic context rather than isolated effects of individual variants. Our results suggest that considering promoter– transcribed region combinations may provide a more informative framework for interpreting PON1-related associations. These disease associations may reflect differences in transcriptional regulation, enzyme stability, or substrate specificity that are not captured by steady-state activity measurements.

The restriction of analyses to individuals aged over 55 years was intended to focus on more established metabolic phenotypes and reduce heterogeneity related to early or transient alterations. This approach may be particularly informative in populations with established metabolic dysfunction but limits generalizability to younger populations.

Several limitations should be noted when interpreting these findings. First, the study was conducted in a single population-based cohort, and replication in independent populations will be important to confirm the observed genotype-dependent associations, particularly for genotype- and haplotype-stratified analyses where the analytical sample was moderate in size. Nevertheless, population structure and cryptic relatedness were addressed through genetic quality control, including PCA-based ancestry filtering and relatedness filtering prior to genetic association analyses. Second, metabolic liver risk was assessed using the Fibrotic NASH Index (FNI), a validated surrogate marker, rather than imaging or histological assessment and HDL-related biology may play a mechanistic role in these associations, particularly because PON1 is an HDL-associated enzyme. Residual confounding by lifestyle factors, medication use and other metabolic indicators cannot be excluded. However, sensitivity analyses indicated that the direction of the main C–A haplotype associations was generally consistent, although some estimates were attenuated after adjustment for additional metabolic variables. This should be interpreted cautiously because HDL cholesterol and AST are components of the FNI score, and other metabolic covariates may lie downstream of dysmetabolic disease or within the biological pathway linking PON1 to metabolic phenotypes rather than independent confounders. Finally, serum PONase activity was the only functional PON1 readout available; additional assays of arylesterase and lactonase activity, HDL functionality, and circulating PON1 protein levels will be needed to clarify the underlying mechanisms.

In conclusion, our findings indicate that PON1 promoter–transcribed region genetic configurations are associated with dysglycemia and metabolic liver risk in a genotype-dependent manner. These associations are not fully captured by steady-state serum PONase activity, suggesting that the functional consequences of PON1 genetic variation may extend beyond basal enzyme activity levels. This attractive hypothesis may help spur replication in independent cohorts and further investigation of the underlying mechanisms.

## Data Availability

The original contributions presented in the study are included in the article/**Supplementary Material**. Further inquiries can be directed to the corresponding authors.
