# Supplementary material for: PON1 haplotypes show genotype-dependent associations with dysglycemia and metabolic liver risk beyond paraoxonase activity
Source: Front Endocrinol (Lausanne). 2026 Jul 7;17:1870186. doi: 10.3389/fendo.2026.1870186 (PMC13385122; doi:10.3389/fendo.2026.1870186)
Supplement: Supplementary file 1 [file DataSheet1.pdf]

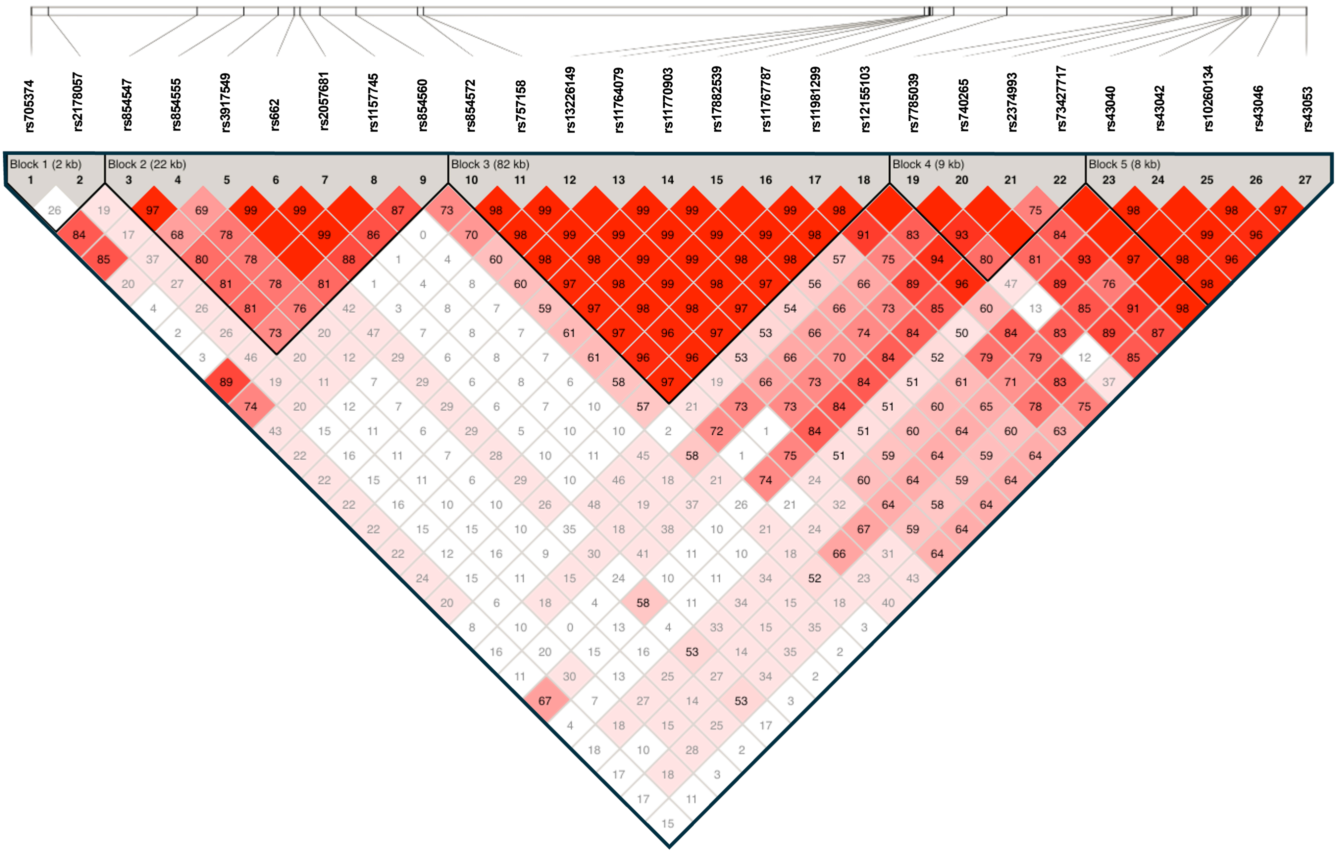

Supplementary Figure 1: Linkage Disequilibrium (LD) map of PON1, PON2 and PON3 genes region, generated by the Haploview software (version 4.1). The LD map was constructed with genotype data of 27 SNPs in the chromosome 7 region (95271366 to 95449748 bp) obtained from 879 individuals of the PREVADIAB2 cohort. Relative SNPs positions in chromosome 7 are depicted in upper bar and respective SNP ID are shown. Pair-wise D' values are shown inside each diamond. The strength of LD is color-coded by LOD score, with red diamonds indicating high LD and white diamonds indicating low LD. The black triangles show 5 LD-blocks defined using the Solid Spine of LD method, with the spine extended when D′ > 0.73. The seven SNPs associated to PONase activity in the GWAS are within LD block 2, while rs854572 identified in the conditional analysis is in LD block 3.
