# Supplementary material for: PON1 haplotypes show genotype-dependent associations with dysglycemia and metabolic liver risk beyond paraoxonase activity
Source: Front Endocrinol (Lausanne). 2026 Jul 7;17:1870186. doi: 10.3389/fendo.2026.1870186 (PMC13385122; doi:10.3389/fendo.2026.1870186)
Supplement: Supplementary file 2 [file DataSheet2.pdf]

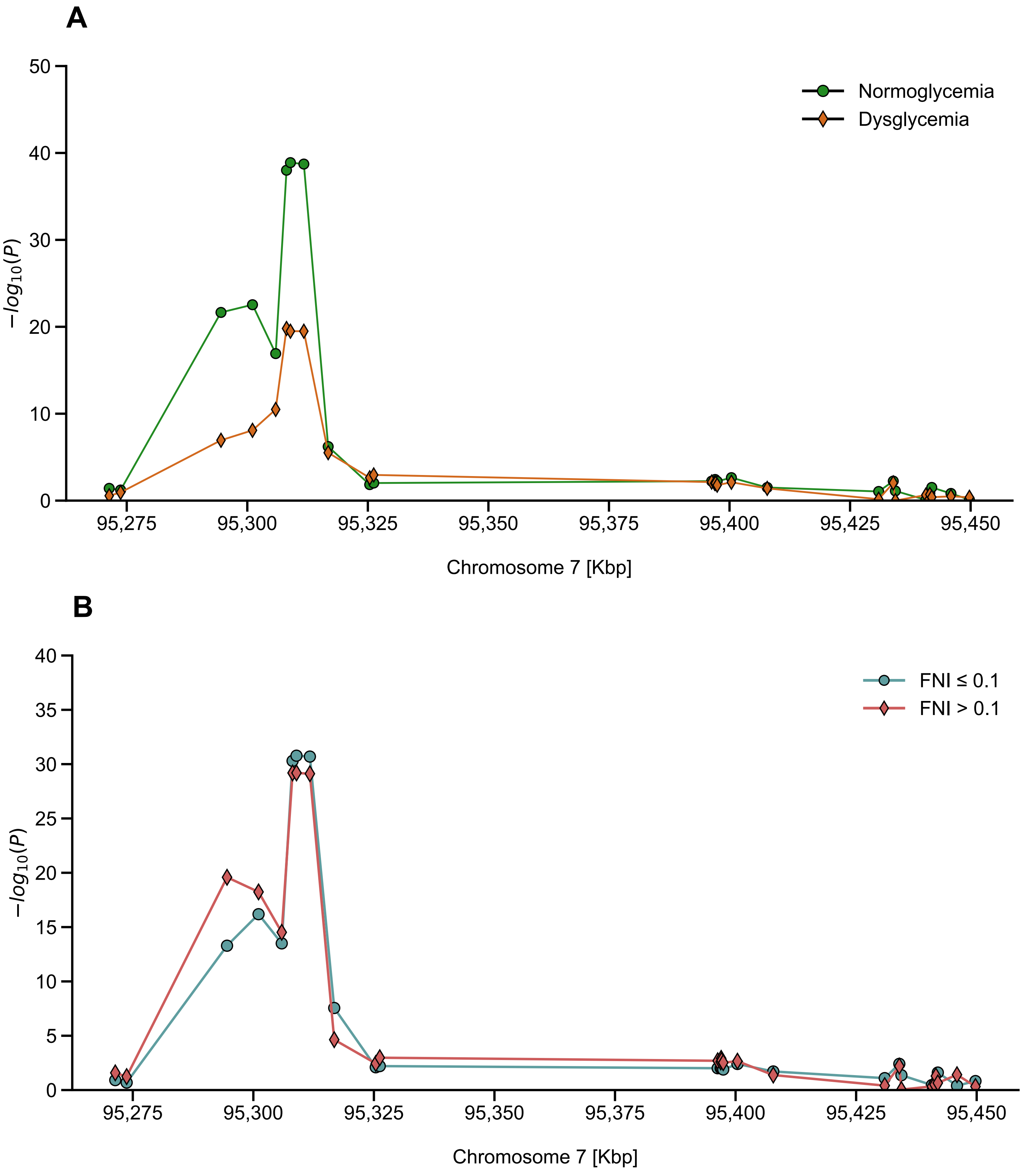

Supplementary Figure 2. Association peak of PONase activity is not affected by glycaemic status or liver dysmetabolism. Stratified association analysis of serum PONase activity in the PON1, PON2 and PON3 gene region. PREVADIAB2 subjects were stratified by (A) glycaemic status into normoglycaemic (n=534, green) and dysglycaemic (n=230, orange) groups, or (B) according to the Fibrotic NASH Index (FNI) into FNI ≤ 0.1 (n=465, blue) and FNI > 0.1 (n=315, red). Plots show the results of association tests (-log10 of P-value) and the genomic position of tested SNPs encompassing the PON1, PON2 and PON3 gene region on chromosome 7 (95,271,366-95,449,748 bp).
