# Supplementary figures and images for: PON1 haplotypes show genotype-dependent associations with dysglycemia and metabolic liver risk beyond paraoxonase activity
Source: Front Endocrinol (Lausanne). 2026 Jul 7;17:1870186. doi: 10.3389/fendo.2026.1870186 (PMC13385122; doi:10.3389/fendo.2026.1870186)

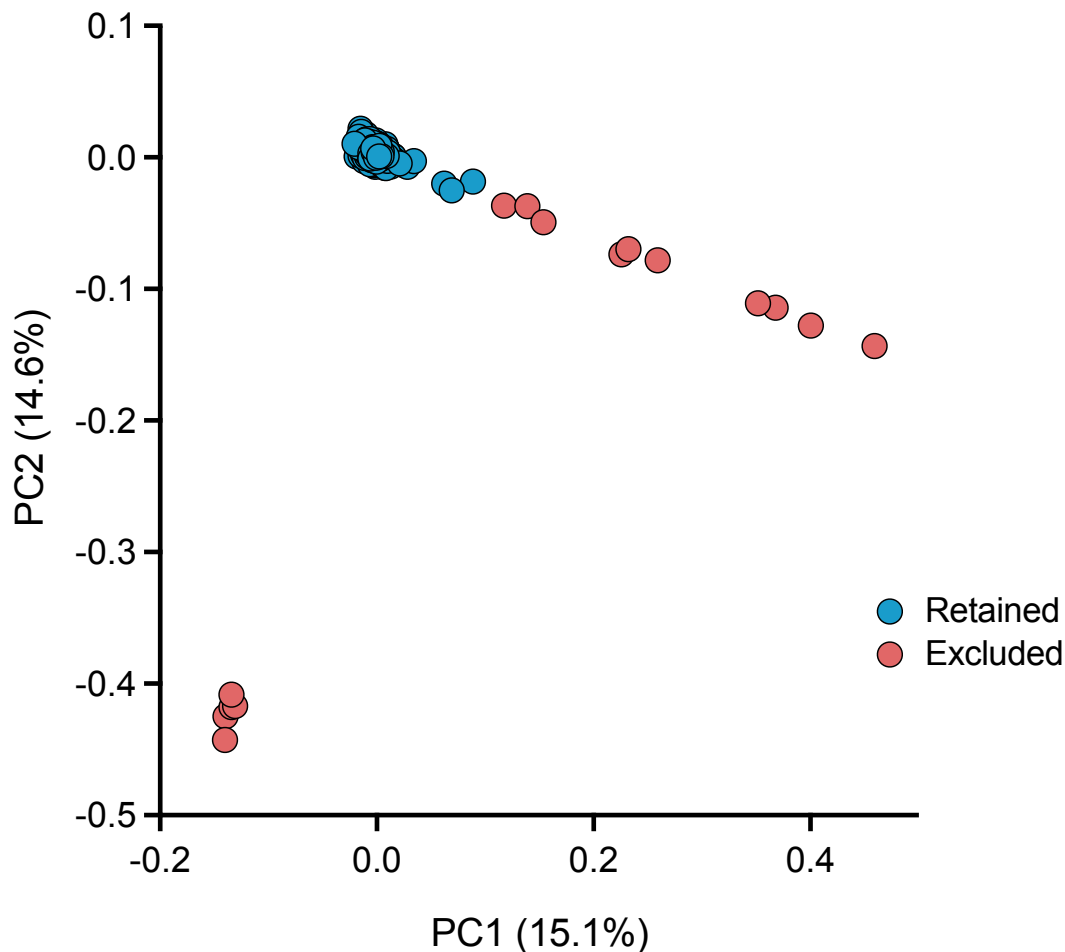

Supplement: Supplementary file 3 [file DataSheet3.pdf]
