# Supplementary material for: PON1 haplotypes show genotype-dependent associations with dysglycemia and metabolic liver risk beyond paraoxonase activity
Source: Front Endocrinol (Lausanne). 2026 Jul 7;17:1870186. doi: 10.3389/fendo.2026.1870186 (PMC13385122; doi:10.3389/fendo.2026.1870186)
Supplement: Supplementary file 4 [file DataSheet4.pdf]

Supplementary Table 1: Association of PONase activity to SNPs in the PON1, PON2 and PON3 genes region, before and after stratification for glycemic status (normoglycemia and dysglycemia) or liver fibrosis risk (FNI ≤ 0.1 and FNI > 0.1).

				All Individuals								Normoglycemia			Dysglycemia			FNI ≤ 0.1			FNI > 0.1		
CHR	BP	Ref. SNP	Ref. All.	n	HWE	MAF	BETA	STAT	*P*	P Adj. Bonf.	P Adj. FDR	n	*P*	P Adj. Bonf.	n	*P*	P Adj. Bonf.	n	*P*	P Adj. Bonf.	n	*P*	P Adj. Bonf.
7	95271366	rs705374	G	784	0.38	0.11	12.18	2.39	1.70E-02	4.59E-01	2.30E-02	533	3.89E-02	1.00E+00	230	2.76E-01	1.00E+00	465	1.22E-01	1.00E+00	314	2.61E-02	7.04E-01
7	95273727	rs2178057	T	782	0.25	0.49	12.26	2.61	9.11E-03	2.46E-01	1.37E-02	532	6.06E-02	1.00E+00	230	1.14E-01	1.00E+00	462	2.05E-01	1.00E+00	315	5.76E-02	1.00E+00
7	95294544	rs854547	G	784	0.77	0.38	45.53	11.78	1.32E-29	3.55E-28	7.11E-29	534	2.23E-22	6.03E-21	229	1.14E-07	3.07E-06	465	5.17E-14	1.40E-12	314	2.60E-20	7.01E-19
7	95301079	rs854555	A	785	0.72	0.37	46.81	12.24	1.27E-31	3.42E-30	8.55E-31	534	2.90E-23	7.82E-22	230	8.15E-09	2.20E-07	465	6.50E-17	1.76E-15	315	5.78E-19	1.56E-17
7	95305887	rs3917549	T	785	0.43	0.18	45.74	11.39	6.84E-28	1.85E-26	3.08E-27	534	1.21E-17	3.28E-16	230	3.25E-11	8.77E-10	465	3.16E-14	8.52E-13	315	3.01E-15	8.14E-14
7	95308134	rs662	C	784	0.38	0.31	61.35	17.69	4.13E-59	1.12E-57	3.72E-58	533	9.71E-39	2.62E-37	230	1.57E-20	4.23E-19	464	5.04E-31	1.36E-29	315	6.45E-30	1.74E-28
7	95308945	rs2057681	G	785	0.43	0.31	61.60	17.79	1.14E-59	3.08E-58	2.17E-58	534	1.30E-39	3.50E-38	230	3.22E-20	8.69E-19	465	1.67E-31	4.51E-30	315	6.45E-30	1.74E-28
7	95311726	rs1157745	T	783	0.39	0.31	61.67	17.76	1.61E-59	4.35E-58	2.17E-58	532	1.89E-39	5.11E-38	230	3.22E-20	8.69E-19	464	2.01E-31	5.44E-30	314	7.63E-30	2.06E-28
7	95316772	rs854560	T	784	0.94	0.39	-29.03	-7.07	3.52E-12	9.50E-11	1.36E-11	533	6.05E-07	1.63E-05	230	3.08E-06	8.31E-05	465	2.79E-08	7.54E-07	314	2.30E-05	6.22E-04
7	95325384	rs854572	C	784	0.23	0.40	16.48	3.91	9.92E-05	2.68E-03	1.91E-04	533	1.45E-02	3.90E-01	230	2.38E-03	6.42E-02	464	8.00E-03	2.16E-01	315	3.56E-03	9.61E-02
7	95326216	rs757158	T	785	0.39	0.37	17.74	4.30	1.97E-05	5.31E-04	6.64E-05	534	9.51E-03	2.57E-01	230	1.11E-03	2.99E-02	465	6.23E-03	1.68E-01	315	1.05E-03	2.85E-02
7	95396288	rs13226149	A	784	0.63	0.23	16.39	3.97	7.77E-05	2.10E-03	1.75E-04	534	5.80E-03	1.57E-01	229	7.38E-03	1.99E-01	465	9.77E-03	2.64E-01	314	2.02E-03	5.46E-02
7	95396917	rs11764079	T	785	0.57	0.23	16.16	3.92	9.64E-05	2.60E-03	1.91E-04	534	5.80E-03	1.57E-01	230	1.01E-02	2.74E-01	465	9.77E-03	2.64E-01	315	2.54E-03	6.85E-02
7	95397015	rs11770903	G	785	0.57	0.23	16.68	4.05	5.66E-05	1.53E-03	1.53E-04	534	3.70E-03	9.99E-02	230	1.01E-02	2.74E-01	465	9.77E-03	2.64E-01	315	1.22E-03	3.29E-02
7	95397096	rs17882539	A	785	0.64	0.23	16.36	3.97	7.71E-05	2.08E-03	1.75E-04	534	5.65E-03	1.53E-01	230	1.01E-02	2.74E-01	465	9.55E-03	2.58E-01	315	1.91E-03	5.16E-02
7	95397441	rs11767787	C	782	0.70	0.23	15.74	3.81	1.49E-04	4.03E-03	2.52E-04	533	6.08E-03	1.64E-01	228	1.67E-02	4.52E-01	464	1.29E-02	3.48E-01	313	3.01E-03	8.12E-02
7	95400389	rs11981299	A	779	0.92	0.21	17.30	4.17	3.36E-05	9.07E-04	1.01E-04	529	2.29E-03	6.19E-02	229	7.73E-03	2.09E-01	461	3.95E-03	1.07E-01	313	2.19E-03	5.92E-02
7	95407819	rs12155103	A	782	0.67	0.20	13.23	3.11	1.92E-03	5.17E-02	3.04E-03	533	3.10E-02	8.37E-01	228	3.93E-02	1.00E+00	464	1.95E-02	5.26E-01	313	4.16E-02	1.00E+00
7	95430930	rs7785039	T	784	0.85	0.22	-7.67	-1.83	6.71E-02	1.00E+00	7.88E-02	534	8.89E-02	1.00E+00	229	6.88E-01	1.00E+00	465	8.01E-02	1.00E+00	314	3.90E-01	1.00E+00
7	95433958	rs740265	G	779	0.26	0.23	15.87	3.86	1.23E-04	3.31E-03	2.21E-04	530	5.69E-03	1.54E-01	228	9.18E-03	2.48E-01	462	3.89E-03	1.05E-01	312	6.23E-03	1.68E-01
7	95434390	rs2374993	G	782	1.00	0.15	-7.26	-1.60	1.10E-01	1.00E+00	1.23E-01	532	7.74E-02	1.00E+00	229	9.99E-01	1.00E+00	463	4.30E-02	1.00E+00	314	9.01E-01	1.00E+00
7	95440708	rs73427717	A	782	0.82	0.18	-1.79	-0.41	6.84E-01	1.00E+00	6.84E-01	531	8.24E-01	1.00E+00	230	2.28E-01	1.00E+00	464	3.47E-01	1.00E+00	313	4.61E-01	1.00E+00
7	95441264	rs43040	C	782	0.92	0.22	-5.48	-1.31	1.92E-01	1.00E+00	2.07E-01	532	1.62E-01	1.00E+00	229	9.02E-01	1.00E+00	464	2.98E-01	1.00E+00	313	3.13E-01	1.00E+00
7	95441538	rs43042	A	784	0.59	0.33	-8.50	-2.07	3.88E-02	1.00E+00	4.99E-02	533	1.40E-01	1.00E+00	230	1.99E-01	1.00E+00	465	2.39E-01	1.00E+00	314	5.10E-02	1.00E+00
7	95441941	rs10260134	T	781	0.72	0.25	10.26	2.51	1.24E-02	3.34E-01	1.76E-02	532	3.06E-02	8.27E-01	228	4.05E-01	1.00E+00	462	2.46E-02	6.63E-01	314	2.22E-01	1.00E+00
7	95445918	rs43046	A	782	0.88	0.33	-7.84	-1.91	5.72E-02	1.00E+00	7.02E-02	532	1.50E-01	1.00E+00	229	3.25E-01	1.00E+00	464	3.96E-01	1.00E+00	313	3.87E-02	1.00E+00
7	95449748	rs43053	C	778	0.73	0.41	-3.04	-0.71	4.80E-01	1.00E+00	4.98E-01	530	7.90E-01	1.00E+00	227	4.14E-01	1.00E+00	461	1.49E-01	1.00E+00	312	4.71E-01	1.00E+00

Results are based on a dominant genetic model adjusted for age, sex, and BMI. CHR: chromosome; BP: base pair position (GRCh38.p14); Ref. SNP: reference SNP ID; Ref. All.: reference allele; n: number of subjects; HWE: Hardy–Weinberg equilibrium p-value; MAF: minor allele frequency; BETA: regression coefficient; STAT: t-statistic; *P*: nominal p-value; P Adj. Bonf.: Bonferroni-corrected p-value for the full cohort; P Adj. FDR: False Discovery Rate adjusted p-value (Benjamini–Hochberg method); FNI: Fibrotic NASH Index.
