# Supplementary material for: PON1 haplotypes show genotype-dependent associations with dysglycemia and metabolic liver risk beyond paraoxonase activity
Source: Front Endocrinol (Lausanne). 2026 Jul 7;17:1870186. doi: 10.3389/fendo.2026.1870186 (PMC13385122; doi:10.3389/fendo.2026.1870186)
Supplement: Supplementary file 5 [file DataSheet5.pdf]

Supplementary Table 2: Association of PONase activity to SNPs in the PON1, PON2 and PON3 genes region using an additive genetic model before and after conditioning for rs2057681.

CHR	BP	Ref. SNP	Ref. All.	n	HWE	MAF	STAT	Additive Model *P*	Additive Model *P* Adj. Bonf.	Additive Model *P* Adj. FDR.	Conditional Model *P*	Conditional Model P Adj. Bonf.	Conditional Model P Adj. FDR
7	95271366	rs705374	G	784	0.38	0.11	2.62	8.90E-03	2.40E-01	1.20E-02	1.12E-02	2.69E-01	2.44E-02
7	95273727	rs2178057	T	782	0.25	0.49	3.10	2.02E-03	5.45E-02	2.87E-03	3.97E-01	1.00E+00	4.99E-01
7	95294544	rs854547	G	784	0.77	0.38	10.69	5.70E-25	1.54E-23	2.57E-24	4.99E-01	1.00E+00	5.70E-01
7	95301079	rs854555	A	785	0.72	0.37	11.27	2.15E-27	5.81E-26	1.45E-26	1.62E-01	1.00E+00	2.42E-01
7	95305887	rs3917549	T	785	0.43	0.18	10.70	4.92E-25	1.33E-23	2.57E-24	4.04E-01	1.00E+00	4.99E-01
7	95308134	rs662	C	784	0.38	0.31	15.89	1.87E-49	5.05E-48	1.68E-48			
7	95308945	rs2057681	G	785	0.43	0.31	16.01	4.52E-50	1.22E-48	6.10E-49			
7	95311726	rs1157745	T	783	0.39	0.31	16.02	3.96E-50	1.07E-48	6.10E-49			
7	95316772	rs854560	T	784	0.94	0.39	-9.97	3.96E-22	1.07E-20	1.53E-21	1.45E-04	3.47E-03	7.42E-04
7	95325384	rs854572	C	784	0.23	0.40	4.51	7.44E-06	2.01E-04	2.23E-05	2.89E-08	6.93E-07	6.93E-07
7	95326216	rs757158	T	785	0.39	0.37	4.80	1.92E-06	5.19E-05	6.49E-06	8.92E-08	2.14E-06	1.07E-06
7	95396288	rs13226149	A	784	0.63	0.23	4.22	2.68E-05	7.24E-04	6.04E-05	1.59E-04	3.82E-03	7.42E-04
7	95396917	rs11764079	T	785	0.57	0.23	4.10	4.49E-05	1.21E-03	8.65E-05	2.21E-04	5.31E-03	7.42E-04
7	95397015	rs11770903	G	785	0.57	0.23	4.12	4.20E-05	1.13E-03	8.65E-05	1.75E-04	4.19E-03	7.42E-04
7	95397096	rs17882539	A	785	0.64	0.23	4.07	5.24E-05	1.42E-03	9.44E-05	2.47E-04	5.93E-03	7.42E-04
7	95397441	rs11767787	C	782	0.70	0.23	3.89	1.08E-04	2.91E-03	1.82E-04	2.79E-04	6.71E-03	7.45E-04
7	95400389	rs11981299	A	779	0.92	0.21	4.43	1.08E-05	2.92E-04	2.92E-05	1.90E-04	4.57E-03	7.42E-04
7	95407819	rs12155103	A	782	0.67	0.20	3.13	1.84E-03	4.96E-02	2.78E-03	1.47E-02	3.53E-01	2.95E-02
7	95430930	rs7785039	T	784	0.85	0.22	-2.07	3.89E-02	1.00E+00	4.57E-02	7.59E-01	1.00E+00	7.92E-01
7	95433958	rs740265	G	779	0.26	0.23	4.27	2.19E-05	5.91E-04	5.37E-05	1.09E-02	2.61E-01	2.44E-02
7	95434390	rs2374993	G	782	1.00	0.15	-1.84	6.61E-02	1.00E+00	7.43E-02	4.16E-01	1.00E+00	4.99E-01
7	95440708	rs73427717	A	782	0.82	0.18	-0.05	9.59E-01	1.00E+00	9.59E-01	2.38E-01	1.00E+00	3.36E-01
7	95441264	rs43040	C	782	0.92	0.22	-1.33	1.85E-01	1.00E+00	2.00E-01	1.45E-01	1.00E+00	2.32E-01
7	95441538	rs43042	A	784	0.59	0.33	-2.54	1.12E-02	3.01E-01	1.43E-02	7.41E-01	1.00E+00	7.92E-01
7	95441941	rs10260134	T	781	0.72	0.25	3.12	1.85E-03	5.00E-02	2.78E-03	5.82E-02	1.00E+00	1.07E-01
7	95445918	rs43046	A	782	0.88	0.33	-2.47	1.37E-02	3.70E-01	1.68E-02	8.59E-01	1.00E+00	8.59E-01
7	95449748	rs43053	C	778	0.73	0.41	-0.94	3.46E-01	1.00E+00	3.60E-01	7.29E-02	1.00E+00	1.25E-01

Results obtained after adjustment for age, sex, and BMI. CHR: chromosome; BP: base pair position (GRCh38.p14); Ref. SNP: reference SNP ID; Ref. All.: reference allele; n: number of subjects; HWE: Hardy–Weinberg equilibrium p-value; MAF: minor allele frequency; STAT: t-statistic from the allelic model; *P*: nominal p-value; P Adj. Bonf.: Bonferroni-corrected p-value; P Adj. FDR: False Discovery Rate adjusted p-value (Benjamini–Hochberg method)
