# Supplementary material for: PON1 haplotypes show genotype-dependent associations with dysglycemia and metabolic liver risk beyond paraoxonase activity
Source: Front Endocrinol (Lausanne). 2026 Jul 7;17:1870186. doi: 10.3389/fendo.2026.1870186 (PMC13385122; doi:10.3389/fendo.2026.1870186)
Supplement: Supplementary file 6 [file DataSheet6.pdf]

Supplementary Table 3: Linkage disequilibrium estimates among key PON1 SNPs

SNP pair	Distance	D'	95% CI D'	R^2	LOD
rs662 vs rs2057681	0.8 kb	0.997	0.98–1.00	0.995	347.71
rs2057681 vs rs854572	16.4 kb	0.01	-0.01–0.13	0	0.01
rs662 vs rs854572	17.2 kb	0.019	-0.01–0.14	0	0.02

D': Lewontin's standardised disequilibrium coefficient; CI: Confidence intervals; R^2: squared correlation coefficient; LOD: log of the odds score; kb: kilobases. Confidence intervals for D' were estimated using Haploview v4.1.
