# Supplementary material for: PON1 haplotypes show genotype-dependent associations with dysglycemia and metabolic liver risk beyond paraoxonase activity
Source: Front Endocrinol (Lausanne). 2026 Jul 7;17:1870186. doi: 10.3389/fendo.2026.1870186 (PMC13385122; doi:10.3389/fendo.2026.1870186)
Supplement: Supplementary file 7 [file DataSheet7.pdf]

Supplementary Table 4: GCTA-COJO conditional and joint analysis of PON1 variants associated with serum PONase activity*

SNP	Effect allele	Effect allele freq.	β	SE	P value	Joint β	Joint SE	Joint P value
rs2057681	G	0.308	45.45	2.826	3.52×10^{-58}	45.63	3.304	2.27×10^{-43}
rs854572	C	0.405	12.12	2.855	2.19×10^{-5}	12.61	2.885	1.24×10^{-5}

* β and SE correspond to marginal association estimates from the genome-wide linear regression model for serum PONase activity. Joint β, joint SE, and joint P values were obtained from GCTA-COJO conditional and joint analysis using cohort-specific LD structure. The model was adjusted for age, sex, and BMI.
