# Supplementary material for: PON1 haplotypes show genotype-dependent associations with dysglycemia and metabolic liver risk beyond paraoxonase activity
Source: Front Endocrinol (Lausanne). 2026 Jul 7;17:1870186. doi: 10.3389/fendo.2026.1870186 (PMC13385122; doi:10.3389/fendo.2026.1870186)
Supplement: Supplementary file 8 [file DataSheet8.pdf]

Supplementary Table 5: Haplotype frequencies of rs854572 and rs2057681 allele combinations in the PREVADIAB2 cohort (n=1,758 haplotypes; 879 individuals)

Haplotype	Alelle rs854572	Alelle rs2057681	n	Freq. (%)
G–A	G	A	717	40.8%
C–A	C	A	497	28.3%
G–G	G	G	334	19.0%
C–G	C	G	210	11.9%
