# Supplementary material for: PON1 haplotypes show genotype-dependent associations with dysglycemia and metabolic liver risk beyond paraoxonase activity
Source: Front Endocrinol (Lausanne). 2026 Jul 7;17:1870186. doi: 10.3389/fendo.2026.1870186 (PMC13385122; doi:10.3389/fendo.2026.1870186)
Supplement: Supplementary file 9 [file DataSheet9.pdf]

Supplementary Table 6: Sensitivity analyses of haplotype associations with dysglycemia and elevated FNI after additional covariate adjustment.

Outcome	rs2057681 genotype	Haplotype	Model*	RR / G-A	RR 95% CI	P-value
Dysglycemia	GA+GG	C-A	Age, Sex, BMI	0.678	0.48 - 0.95	**0.02462**
Dysglycemia	GA+GG	C-A	Age, Sex, BMI + Smoker	0.716	0.51 - 1.01	0.05593
Dysglycemia	GA+GG	C-A	Age, Sex, BMI + HDL	0.671	0.48 - 0.94	**0.01924**
Dysglycemia	GA+GG	C-A	Age, Sex, BMI + AST	0.679	0.48 - 0.96	**0.02632**
Dysglycemia	GA+GG	C-A	Age, Sex, BMI + TAG	0.706	0.51 - 0.99	**0.04072**
Dysglycemia	GA+GG	C-A	Age, Sex, BMI + HTA	0.676	0.48 - 0.95	**0.02337**
FNI >0.1	GA+GG	C-A	Age, Sex, BMI	0.739	0.55 - 0.99	**0.04559**
FNI >0.1	GA+GG	C-A	Age, Sex, BMI + Smoker	0.719	0.53 - 0.98	**0.03635**
FNI >0.1	GA+GG	C-A	Age, Sex, BMI + HDL	0.735	0.56 - 0.97	**0.03089**
FNI >0.1	GA+GG	C-A	Age, Sex, BMI + AST	0.848	0.64 - 1.12	0.24535
FNI >0.1	GA+GG	C-A	Age, Sex, BMI + TAG	0.756	0.57 - 1.01	0.06025
FNI >0.1	GA+GG	C-A	Age, Sex, BMI + HTA	0.742	0.55 - 1.00	**0.04865**
FNI >0.1	AA	C-A	Age, Sex, BMI	1.199	1.01 - 1.42	**0.03476**
FNI >0.1	AA	C-A	Age, Sex, BMI + Smoker	1.216	1.02 - 1.44	**0.02621**
FNI >0.1	AA	C-A	Age, Sex, BMI + HDL	1.169	1.00 - 1.37	**0.04829**
FNI >0.1	AA	C-A	Age, Sex, BMI + AST	1.212	1.02 - 1.44	**0.02555**
FNI >0.1	AA	C-A	Age, Sex, BMI + TAG	1.173	0.99 - 1.38	0.05954
FNI >0.1	AA	C-A	Age, Sex, BMI + HTA	1.195	1.01 - 1.41	**0.03805**

* Adjusted relative risks were estimated using Poisson regression. The primary model included age, sex, and BMI. Sensitivity models additionally included smoking, HDL cholesterol, aspartate aminotransferase (AST), triglycerides (TAG), or hypertension (HTA), as indicated. HDL cholesterol and AST are components of the FNI score and were included only as exploratory sensitivity analyses.
